# Electroactive shape memory polyurethane composites reinforced with octadecyl isocyanate-functionalized multi-walled carbon nanotubes

**DOI:** 10.3389/fbioe.2022.964080

**Published:** 2022-07-15

**Authors:** Yadong Sun, Jiachi Teng, Yi Kuang, Shengxiang Yang, Jiquan Yang, Hongli Mao, Zhongwei Gu

**Affiliations:** ^1^ Research Institute for Biomaterials, Tech Institute for Advanced Materials, College of Materials Science and Engineering, Nanjing Tech University, Nanjing, China; ^2^ College of Chemical and Materials Engineering, Zhejiang A&F University, Lin’an, China; ^3^ Nanjing Industry Institute for Advanced Intelligent Equipment, Nanjing, China; ^4^ NJTech-BARTY Joint Research Center for Innovative Medical Technology, Nanjing, China

**Keywords:** shape memory polyurethane, multi-walled carbon nanotubes, octadecyl isocyanate, functionalization, electroactive

## Abstract

Shape memory polymers (SMPs) have a wide range of potential applications in many fields. In particular, electrically driven SMPs have attracted increasing attention due to their unique electrical deformation behaviors. Carbon nanotubes (CNTs) are often used as SMP conductive fillers because of their excellent electrical conductivities. However, raw CNTs do not disperse into the polymer matrix well. This strictly limits their use. In this study, to improve their dispersion performance characteristics in the polymer matrix, hydroxylated multi-walled carbon nanotubes (MWCNT-OHs) were functionalized with octadecyl isocyanate (i-MWCNTs). Polyurethane with shape memory properties (SMPU) was synthesized using polycaprolactone diol (PCL-diol), hexamethylene diisocyanate (HDI), and 1,4-butanediol (BDO) at a 1:5:4 ratio. Then, electroactive shape memory composites were developed by blending SMPU with i-MWCNTs to produce SMPU/i-MWCNTs. The functionalized i-MWCNTs exhibited better dispersibility characteristics in organic solvents and SMPU composites than the MWCNT-OHs. The addition of i-MWCNTs reduced the crystallinity of SMPU without affecting the original chemical structure. In addition, the hydrogen bond index and melting temperature of the SMPU soft segment decreased significantly, and the thermal decomposition temperatures of the composites increased. The SMPU/i-MWCNT composites exhibited conductivity when the i-MWCNT content was 0.5 wt%. This conductivity increased with the i-MWCNT content. In addition, when the i-MWCNT content exceeded 1 wt%, the composite temperature could increase beyond 60°C within 140 s and the temporary structure could be restored to its initial state within 120 s using a voltage of 30 eV. Therefore, the functionalized CNTs exhibit excellent potential for use in the development of electroactive shape memory composites, which may be used in flexible electronics and other fields.

## Introduction

Because of their designable shape fixation and recovery properties, shape memory materials have attracted extensive attention in recent years (Behl and Lendlein, 2007; Hager et al., 2015). Shape memory polymers (SMPs) are low cost and lightweight, and offer the advantages of easy manufacturability, a wide range of shape memory designs, and responsiveness to multiple stimuli (including temperature, current, magnetic field, pH change, solvent, etc.). These properties make SMPs more advantageous than shape memory alloys and shape memory ceramics (Leng et al., 2011; Hu et al., 2012; Hardy et al., 2016; Kim et al., 2021; Du et al., 2022). SMPs are used widely in aerospace, biomedicine, flexible electronics, textile manufacturing and other fields (Gao et al., 2019; Sun et al., 2020; Xia et al., 2021; Sun et al., 2022).

One of the most used SMPs, shape memory polyurethane (SMPU) is usually composed of a hard segment with a high glass transition temperature (T_g_) and a soft segment with a low glass transition temperature (Laza et al., 2020; Liu et al., 2021). The segmented structures are thermodynamically incompatible and exhibit microphase separation on the molecular scale. The hard segment is used as the stationary phase and provides strength to ensure the shape recovery of the material, whereas the soft segment is a reversible phase that serves to deform the material and fix its shape temporarily (Zhu et al., 2008; Liu et al., 2017; Park et al., 2021). However, traditional SMPU produces shape memory effects only when stimulated directly using heat. This often limits its applications. Therefore, stimulation method development studies have been performed. By introducing azobenzene side-chain groups into the SMPU network, Wen et al. achieved shape-reversible bending and straightening of SMPU under light irradiation (Wen et al., 2018). Reza et al. doped nanographene sheets into SMPU to produce conductive composites and demonstrated the ability to restore a temporary SMPU structure to its original shape gradually using stimulation from an external current (Sofla et al., 2019). Among the various stimuli used to drive SMPUs, light driving must be performed in a highly controlled environment and thermal driving requires direct heating (Ur Rehman et al., 2018; Xie et al., 2018). Since its inherent conductivity can cause itself to become joule heated and produce remote control effects, SMPU that responds to electrical stimulation offers substantial advantages (Umair et al., 2019). The simplest way to produce such materials is to introduce highly conductive particles (e.g., graphene, carbon black, carbon nanotubes) into the shape memory polymer matrix (Zhang et al., 2017; Xu et al., 2018; Zheng et al., 2020; Raza et al., 2021). Although graphene has higher thermal conductivity, the conductivity of its composites is still worse than that of carbon nanotube composites at the same particle content (Galindo et al., 2016; Ke et al., 2018). For carbon black, more additives are needed in the composites in order to obtain conductive effect (Rosales et al., 2018).

As a promising class of nanomaterials with excellent mechanical, thermal, and electrical properties, carbon nanotubes (CNTs) are uniquely superior in the context of electroactive SMPU preparation (Mahapatra et al., 2014; Ren et al., 2019; Qu et al., 2022). However, to obtain ideal CNT-polymer composites, one must achieve a uniform CNT dispersion in the polymer matrix and sufficient interfacial adhesion. Both of these are challenging requirements. Because of the strong van der Waals forces between carbon nanotubes, they prefer to entangle or agglomerate into bundles, and fail to transfer loads from the polymer matrix (Sahoo et al., 2010; Rahmat et al., 2011; Chazot and Hart, 2019). To overcome this problem, it is crucial to modify the CNT surfaces such that they do not aggregate in the polymer matrix (Kim et al., 2009; Zahedi and Amraee, 2018).

In this study, octadecyl isocyanate that can react with hydroxyl or carboxyl groups on CNT surfaces to form carbamate bonds without complex reaction conditions was used to functionalize hydroxylated multi-walled carbon nanotubes (MWCNT-OH) and the dispersion properties of the modified i-MWCNTs were analyzed in various solvents. Then, SMPU/i-MWCNT composites with various i-MWCNT contents were prepared via solution casting. The physicochemical properties and electrically driven shape memory performance characteristics of the resulting materials were then studied in detail.

## Experimental

### Materials

Polycaprolactone-diol (PCL-diol, Mn = 4,000 g/mol) was purchased from Shenzhen Guanghuaweiye Co. Ltd (China). 1,4-butanediol (BDO, purity ≥ 98%), hexamethylene diisocyanate (HDI, purity ≥ 99%), toluene (purity ≥ 99%), dimethylformamide (DMF, purity ≥ 99%), stannous octoate (Sn(Oct)_2_, purity ≥ 96%), and hydroxylated multi-walled carbon nanotubes (MWCNT-OH, Length: 20–30 nm) were supplied by Aladdin (China). Octadecyl isocyanate was purchased from Aldrich (United States). Dichloromethane (DCM, purity ≥ 99%) and tetrahydrofuran (THF, purity ≥ 99%) were obtained from 3A Chem (China).

### Synthesis of i-MWCNTs

**FIGURE 1:**
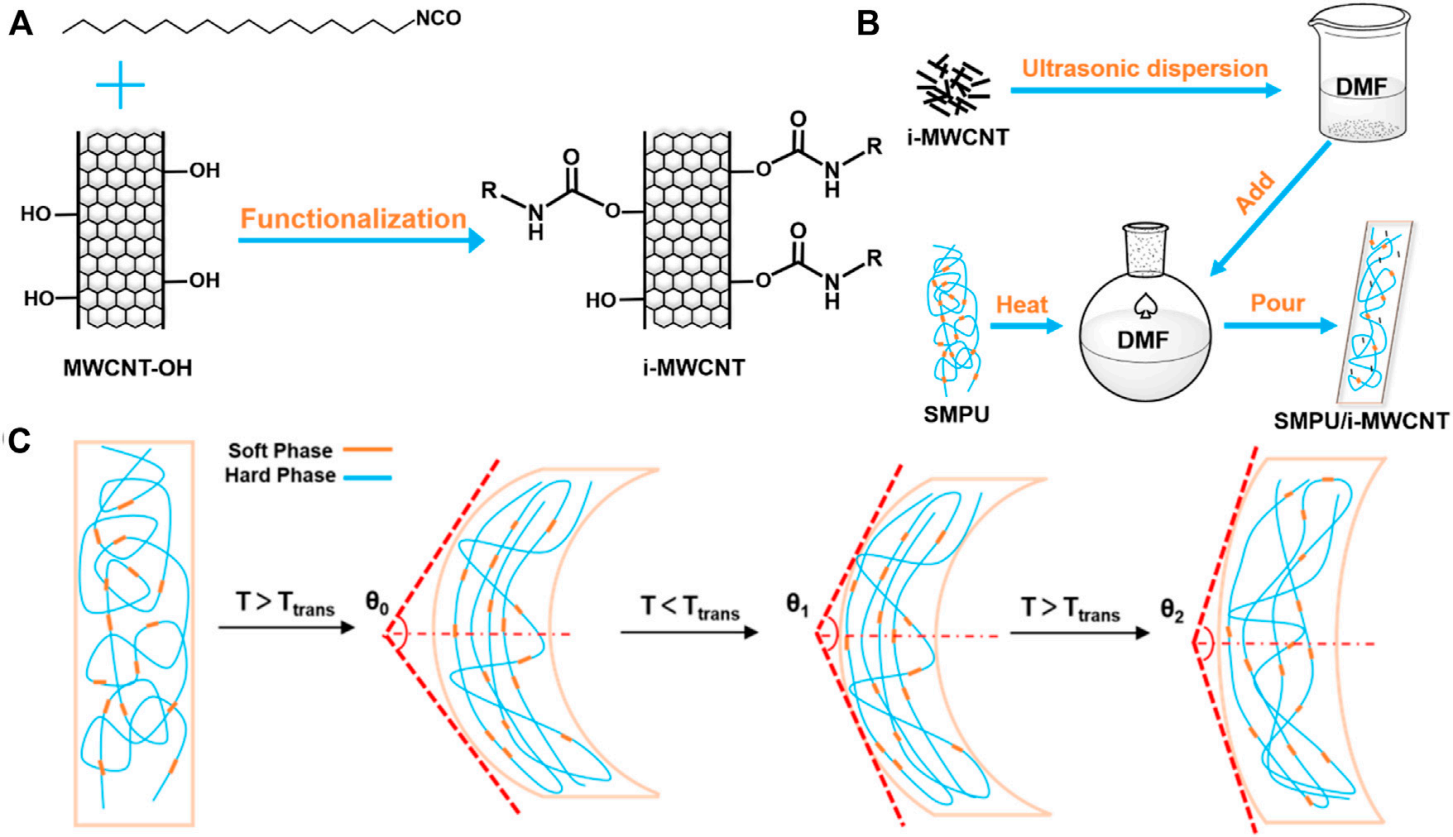
**(A)** Synthesis of i-MWCNT. **(B)** Preparation of SMPU/i-MWCNT composites. **(C)** Schematic illustration of the shape memory behavior test.

First, 1.00 g of MWCNT-OH was dispersed in 40 ml of anhydrous DMF solution via ultrasonic dispersion. Then, 40 ml of anhydrous DMF solution with 3.00 g of octadecyl isocyanate dissolved in it was mixed with the MWCNT-OH solution and stirred at room temperature (RT) for 8 h under nitrogen. The -OH groups on the MWCNT-OH surfaces reacted with the -NCO groups within octadecyl isocyanate to form urethane linkages (Figure 1A). The product was dried under vacuum at 80°C after being centrifuged and washed with dichloromethane three times.

### Synthesis of shape memory polyurethane

SMPU was synthesized gradually via pre-polymerization using a PCL:HDI:BDO molar ratio of 1:5:4. First, 10.00 g of PCL was placed in a three-port flask and dried under vacuum at 100°C for 4 h. When the temperature decreased to RT, 35 ml of anhydrous toluene was injected into the flask, which was then heated to 65°C and stirred for 30 min until the PCL was dissolved completely in the anhydrous toluene. Then, 2.10 g of HDI and two drops of stannous octanoate (0.1 wt% of PCL) were added to the flask. The mixture was heated to 90°C and stirred under nitrogen for 2.5 h. Finally, 1.80 g of BDO was added to the reaction mixture to enable chain extension and thus increase the molecular weight of the polymer. The resulting mixture was stirred quickly for 20 min. The product was transferred into a vacuum drying oven and dried at 80°C for 1 d. The hard segment content of the prepared SMPU was 23% (Nejad et al., 2019; Nouri et al., 2020).

### Preparation of SMPU/i-MWCNT composites

Polyurethane matrix composites that contained various quantities of functionalized carbon nanotubes (0 wt%, 0.1 wt%, 0.5 wt%, 1 wt%, and 2 wt%) were prepared via the solution mixed casting method. The resulting materials were named PU, PU-0.1, PU-0.5, PU-1, and PU-2, respectively (Figure 1B). Briefly, 4.00 g of SMPU was dissolved in 40 ml of DMF and stirred at 85°C to produce a transparent polymer solution. Separately, i-MWCNTs were dispersed in DMF via ultrasonic dispersion for 2 h and then mixed with the SMPU solution, stirred for 20 min, and dried quickly for 30 min. The resulting SMPU/i-MWCNT composites were poured into PTFE molds and dried under vacuum at 80°C for 24 h. The SMPU/MWCNT-OH composite with a raw MWCNT-OH content of 1 wt% was used as a negative control.

### Characterization

#### Fourier transform infrared spectroscopy (FT-IR)

The infrared spectra of the samples were measured using a Fourier infrared spectrometer (Nicolet iS5, Thermo Scientific). Each sample was scanned 32 times in the 500–4,000 cm^−1^ range. The hydrogen bonding indexes of the C=O functional groups (HBI_(C=O)_, %) within SMPU and SMPU/i-MWCNT composites with different i-MWCNT contents were determined using the method and equation (Eq. 1) mentioned in previous studies (Babaie et al., 2019).

$$HBI_{\left(C=O\right)}\frac{A_{\left(C=O\right)}^{b}}{A_{\left(C=O\right)}^{b}+A_{\left(C=O\right)}^{nb}}\times 100\%$$ (1)

where $A_{\left(C=O\right)}^{b}$ and $A_{\left(C=O\right)}^{nb}$ are the areas of the free and hydrogen-bonded carbonyl functional bands.

### X-ray diffraction

An X-ray diffractometer (Smartlab 9 kW, Japan) equipped with a scintillation counter, CuK α radiation (*λ* = 0.1540 nm) with an accelerating voltage of 40 kV, and a current of 40 mA was used to record the XRD patterns of the samples. The crystal size and crystallinity equations used with SMPU and SMPU/i-MWCNT composites with various i-MWCNT contents are Eqs. 2, 3, respectively (Eyvazzadeh Kalajahi et al., 2017).

$$L_{hkl}=\frac{K\lambda }{\beta_{hkl}\times cos\theta_{hkl}}$$ (2)

where L_hkl_, β_hkl_, and θ_hkl_ represent the crystal size, diffraction full-width at half-maximum (FWHM), and (hkl) plane Bragg angle, respectively. *λ* and *K* are the X-ray wavelength (0.1540 nm) and Scherrer constant (0.9), respectively.

$$X_{c}=\frac{A_{110}+A_{111}+A_{200}}{A_{110}+A_{111}+A_{200}+A_{ah}}\times 100\%$$ (3)

where A_110_, A_111_, and A_200_ represent the areas of the crystalline diffraction peaks and A_ah_ is attributed to the amorphous halo noted in the XRD patterns.

### Laser confocal Raman spectroscopy

The i-MWCNT structural order was characterized using a laser confocal Raman spectrometer (Horiba Evolution, Japan) with a laser wavelength of 632.8 nm and a resolution of 3 cm^−1^. C, N, and O in the i-MWCNT were analyzed using a k-alpha photoelectron spectrometer (Thermo Scientific).

### Differential scanning calorimetry

The DSC curves of PCL, SMPU, and several SMPU/i-MWCNT composites with various i-MWCNT contents were obtained under nitrogen using a differential scanning calorimeter (DSC 200-F3, Netzsch). First, each sample was heated to 200°C at a heating rate of 15°C/min and maintained at a constant temperature for 2 min to eliminate the its thermal history of the sample. Then, each sample was cooled to −80 °C at a cooling rate of 15°C/min and maintained at a constant temperature for 2 min. Finally, each sample was heated to 200°C again at a heating rate of 15°C/min. The degree of sample crystallinity, melting temperature, and glass transition temperature were evaluated using data from the first cooling and the second heating cycles. The soft segment crystallinity was calculated as shown in equation Eq. 4 (Nouri et al., 2020).

$$X_{c}=\frac{\Delta H_{ss}}{136\times 1-G-HS}\times 100\%$$ (4)

where 136 and *ΔH*_*ss*_ are the heat of fusion of 100% crystalline PCL-diol and the soft segments in the relevant samples. G and HS represent the masses of graphene nanosheets and hard segments in the composites.

### Scanning electron microscopy

The fractured surface micromorphologies of SMPU and SMPU/i-MWCNT composites with various i-MWCNT contents after tensile fracture were analyzed using a scanning electron microscope (JSM-IT200). Before scanning, the specimen sections were sprayed with gold at a voltage of 2.5 kV.

### Thermal stability

The thermal stability characteristics of i-MWCNT, SMPU, and SMPU/i-MWCNT composites with various i-MWCNT contents were tested using a synchronous thermal analyzer (STA 449-F3, Germany). Briefly, the samples were dried and their weight loss was determined using a heading rate of 10°C/min under nitrogen.

### Electrical conductivity

The electrical conductivities of the SMPU and SMPU/i-MWCNT composites with various i-MWCNT contents were measured via a four-probe technique using a surface resistance detector (RHA-FT-331, China). A voltage of 30 V was applied to the samples using two electrodes located 10 mm apart and the surface temperature of the specimen was recorded via a laser digital infrared thermometer.

### Mechanical properties

A universal tensile testing machine (Instron 5,943, United States) was used to test the tensile strengths of SMPU and several SMPU/i-MWCNT composites with various i-MWCNT contents at RT. The gauge length of the extensometer was 25 mm and the tensile rate was 10 mm/min. Each sample was tested three times and the average value was recorded.

### Shape memory properties

A prepared rectangular spline was used to test the shape memory fixation (R_f_) and recovery (R_r_) rates. As shown in Figure 1C, the test process followed a sequence that included heating deformation, cooling fixation, and heating recovery. First, the spline was placed in 60°C water for 10 min. Then, the spline was bent at an angle of θ_0_ using an external force, and placed in ice water for 5 min. Next, the angle was measured again and recorded as θ_1_. Finally, the spline was heated in 60°C water for 10 min, and the resulting angle was recorded as θ_2_. The R_f_ and R_r_ of the specimen were calculated using Eqs. 5, 6 (Moghim et al., 2018).

$$R_{f}=\frac{180-\theta_{1}}{180-\theta_{2}}\times 100\%$$ (5)

$$R_{r}=\frac{\theta_{2}-\theta_{1}}{180-\theta_{1}}\times 100\%$$ (6)

## Results and discussion

### i-MWCNT preparation and characterization

**FIGURE 2:**
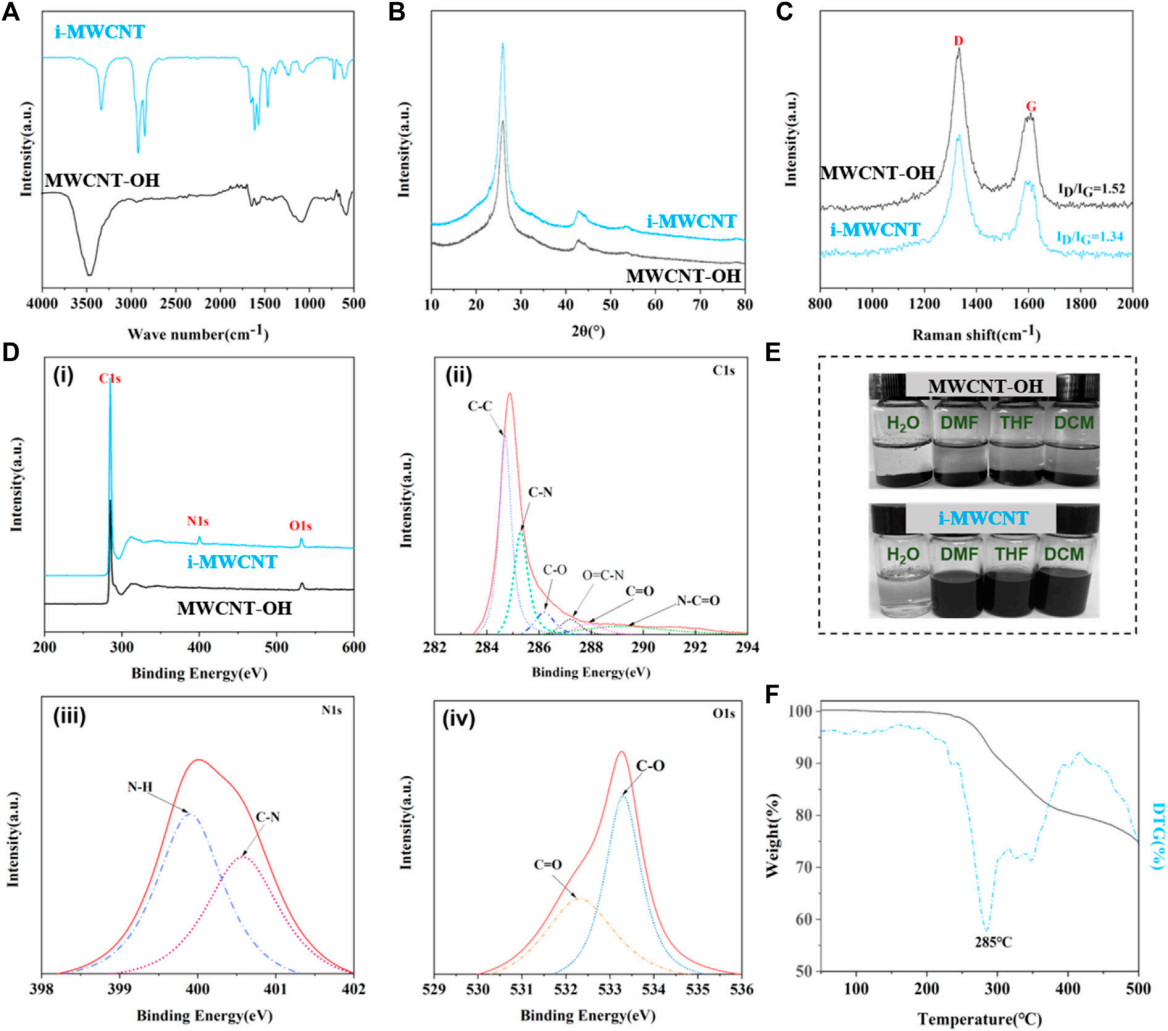
**(A)** FT-IR, **(B)** XRD, and **(C)** Raman spectra of MWCNT-OH and i-MWCNT. **(D)** XPS wide-scan spectra of MWCNT-OH and i-MWCNT (i: high-resolution elements, ii: C1s, iii: O1s, iv: N1s). **(E)** Photos of MWCNT-OH and i-MWCNT dispersed in various solvents. **(F)** i-MWCNT TG-DTG curves.

Figure 2A shows the FT-IR spectra of the hydroxylated MWCNTs (MWCNT-OHs) and the octadecyl isocyanate-functionalized MWCNTs (i-MWCNTs). In the MWCNT-OH spectrum, the characteristic peak at 3,450 cm^−1^ belongs to the -OH stretching vibration. In the i-MWCNT spectrum, the absorption peaks at 3,340 cm^−1^, 1,614 cm^−1^, 1,575 cm^−1^, and 1,220 cm^−1^ correspond to the N-H stretching vibration, the NH-CO stretching vibration, the N-H bending vibration, and the C-N stretching vibration, respectively. The disappearance of the characteristic hydroxyl peak and emergence of a characteristic carbamate group peak, as well as the absence of octadecyl ester characteristic peaks at 2,271 cm^−1^ and 1,354 cm^−1^ in the i-MWCNT spectrum, combine to indicate that octadecyl isocyanate is successfully grafted onto the MWCNT-OH surface (Zhao et al., 2004; Deng et al., 2007; Lopes et al., 2017; Salam and Burk, 2017).

The MWCNT-OH and i-MWCNT XRD patterns are shown in Figure 2B. Two of the diffraction peaks present are characteristic of carbon nanotubes: the (002) diffraction peak (the strongest carbon peak with 2θ = 26° and d = 0.45 nm) and the (100) diffraction peak (the secondary carbon diffraction peak with 2θ = 42.6° and d = 0.21 nm) appear at the same positions in the two curves. This suggests that the MWCNT-OH structure does not change upon modification with octadecyl isocyanate. Thus, the functional modification process does not destroy the original MWCNT crystal structure (Bi et al., 2020).

To analyze the interactions on the octadecyl isocyanate-modified nanotube surface further, the structure and morphology of MWCNT-OH and i-MWCNT were analyzed via Raman spectroscopy. Figure 2C shows that both MWCNT-OH and i-MWCNT exhibit two D and G peaks. This indicates that the overall structure of the nanotubes does not change upon modification. The I_D_/I_G_ value for MWCNT-OH (1.52) is greater than that for i-MWCNT (1.34). This suggests an increase in the average size of the C-C sp^3^ domain after functionalization. This may be because modification with octadecyl isocyanate introduces long aliphatic chains on the nanotube surface (Tamore et al., 2019).

Figure 2D(i) shows XPS wide-scan spectra of MWCNT-OH and i-MWCNT. The full i-MWCNT XPS spectrum exhibits not only C1s and O1s optoelectronic spectra, but also a new N1s optoelectronic peak at 401.2 eV, that is, not present in the corresponding MWCNT-OH spectrum. After peak splitting fitting, new C-N and O=C-N optoelectronic peaks appear at 285.3 and 287.2 eV, respectively, in the C1s XPS spectrum of i-MWCNT (Figure 2D(ii)). Two new photoelectric peaks at 400.1 and 400.9 eV in the N1s XPS spectrum correspond to the characteristic peaks of -NH- and C-N, respectively (Figure 2D(iii)). Two optoelectronic peaks from C=O (522.3 eV) and C-O (533.3 eV) are observed in the N1s XPS spectrum of i-MWCNT (Figure 2D(iv)). The results indicate the presence of amide (-CO-NH-) and carbamate (-NH-COO-) groups in i-MWCNT. This further demonstrates that octadecyl isocyanate is grafted onto the i-MWCNT surface (Zhao et al., 2004; Lopes et al., 2017; Bi et al., 2020).

Figure 2E shows photos of MWCNT-OH and i-MWCNT samples dispersed via ultrasound in various solvents after standing for 1 d. The MWCNT-OH sample exhibits poor dispersion in four solvents, while i-MWCNT functionalized with octadecyl isocyanate exhibits a perfect dispersion effect in the organic solvents. The i-MWCNT surface is grafted with long-chain molecules that can effectively prevent direct contact between carbon nanotube skeletons and weaken the van der Waals force between the carbon nanotubes. Moreover, -NCO reacts with the -OH groups on the MWCNT-OH surface to form urethane linkages. Therefore, i-MWCNTs can disperse well in organic reagents (Kim et al., 2009; Salam and Burk, 2017).

A TGA test was performed to investigate the thermal stability of i-MWCNT. TG and DTG curves were obtained for i-MWCNT (Figure 2F). The i-MWCNTs decompose as the temperature increases, reaching their maximum decomposition rate at 285°C. The boiling point of octadecyl isocyanate is 173°C. If octadecyl isocyanate is only adsorbed onto the carbon nanotube surface, it is expected to volatilize. Therefore, the peak in the DTG curve corresponds to the decomposition of long alkyl chains grafted onto the i-MWCNT surface. After this point, the decomposition rate decreases gradually, indicating that oxygen-containing groups on the i-MWCNT surface also begin to decompose gradually (Zhao et al., 2004; Deng et al., 2007; Lopes et al., 2017).

### Preparation of SMPU/i-MWCNT composites

**FIGURE 3:**
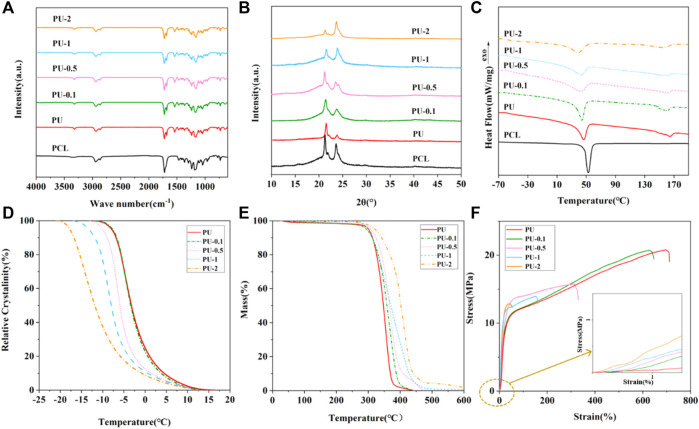
**(A)** FT-IR, **(B)** XRD, and **(C)** DSC curves of PCL, SMPU, and SMPU/i-MWCNT composites with various i-MWCNT contents. **(D)** Relative crystallinity evolution versus temperature, **(E)** TG curves, and **(F)** stress-strain behaviors of SMPU and SMPU/i-MWCNT composites with various i-MWCNT contents at RT.

The FT-IR spectra of PCL, SMPU, and SMPU/i-MWCNT composites with various i-MWCNT contents are shown in Figure 3A. The relatively wide absorption band at 3,350 cm^−1^ in the PCL spectrum corresponds to the -OH group. The absorption peaks at 2,945 cm^−1^, 2,864 cm^−1^, 1,236 cm^−1^, and 962 cm^−1^ are C-H vibration peaks from methylene groups within PCL. The strong absorption peak at 1720 cm^−1^ is attributed to the characteristic absorption peak of the non-hydrogen bonded esoteric group (C=O). Moreover, since PCL-diol lacks proton donor groups, no peak is observed at 1,680 cm^−1^.

In the spectrum of pure SMPU, the absorption peaks at 3,319 cm^−1^ and 1,539 cm^−1^ correspond to the N-H stretching vibration peak and the bending vibration peak from the urethane linkage, respectively. The N-H absorption peak that indicates a lack of involvement in hydrogen bond formation does not appear at 3,378–3,400 cm^−1^. This indicates that all N-H groups are involved in the formation of hydrogen bonds with C=O. This may be caused by the presence of proton acceptor groups (C=O) in both the hard and soft polyurethane segments. The absorption peaks from non hydrogen-bonded free C=O and C=O, that is, hydrogen bonded with N-H appear at 1725 cm^−1^ and 1,684 cm^−1^, respectively. Two absorption peaks are observed for the C-O in the soft segment at 1,156 cm^−1^ and 1,064 cm^−1^. These are the absorption peaks from C-O without and with hydrogen bonding. The disappearance of the -OH contraction vibration peak at 3,450 cm^−1^ and the contraction vibration peak from the isocyanate group (N=C=O) at 2,250–2,274 cm^−1^ indicate the successful synthesis of polyurethane (Sofla et al., 2019; Ren et al., 2019; Nejad et al., 2019).

The SMPU and SMPU/i-MWCNT composite wave peak positions do not change. This indicates that infiltration of i-MWCNTs into SMPU does not change its structure. However, the presence of i-MWCNT has some influence on the hydrogen-bonding group content of SMPU. The hydrogen bond carbonyl index (HBI_(C=O)_, %) of the samples is shown in Table 1. The results indicate that the addition of i-MWCNT reduces the HBI_(C=O)_ contents of the SMPU composites (Sofla et al., 2019; Nejad et al., 2019). It may be owing to the formation of hydrogen bonds between the polyurethane bonds formed in the modified i-MWCNTs and the hard segments of the polyurethane. The carbonyl groups within the carbamate groups in SMPU can form intermolecular hydrogen bonds with amino groups. To verify the accuracy of the calculation results, an ideal polyurethane chain with a PCL:HDI:BDO ratio of 1:5:4 was analyzed statistically. The molecular weight of PCL is 4,000 g/mol, and the molecular weight of its repeat unit is 114 g/mol. Thus, each PCL segment contains approximately 34 carbonyl bases. HDI and BDO form nine carbonyl groups in SMPU. Ideally, the polyurethane chain should contain 43 carbonyl groups. The FT-IR spectrum shows that all such groups are involved in the reaction. Therefore, the HBI_(C=O)_ content in the ideal SMPU is 21.0%, which is consistent with the calculation results in Table 1 (Nouri et al., 2020).

**TABLE 1:** The hydrogen bonding indexes HBI_(C=O)_, crystal sizes of planes (110) and (200) (L_110_ and L_200_), and crystallinity contents [Xc (%)] of PCL, SMPU, and SMPU/i-MWCNT composites with various i-MWCNT contents.

Sample	$A_{\left(C=O\right)}^{b}$	$A_{\left(C=O\right)}^{nb}$	$HBI{\;}_{\left(C=O\right)}$ (%)	L_110_ (nm)	L_200_ (nm)	X_c_ (%)
PCL	—	—	—	34.12	27.54	56.36
PU	4.25	13.40	24.08	19.86	24.34	31.56
PU-0.1	3.55	12.18	22.57	27.38	22.81	30.24
PU-0.5	3.22	12.07	21.06	26.56	15.52	22.18
PU-1	3.18	11.65	21.44	25.89	16.31	21.81
PU-2	3.45	12.37	21.81	25.95	15.41	20.73

The crystal structures of the samples were calculated via the XRD method, and the results are shown in Figure 3B. PCL-diol has three main diffraction peaks at 2θ = 21.6°, 22.2°, and 24.0°. The same peaks also appear in the XRD spectra of the SMPU and SMPU/i-MWCNT composites. This indicates that the crystal structures of the SMPU and SMPU/i-MWCNT composites are caused by the PCL soft segment. The crystal sizes and crystallinities of the samples are calculated and the results are shown in Table 2. Different from the structure of PCL-diol, the anchoring effects and the presence of hard segments led to the formation of smaller crystals in the SMPU structure. Sample crystallinity decreases as the i-MWCNT content increases. This was because the addition of i-MWCNT may hinder the orderly arrangement of PU segments during crystallization (Hashmi et al., 2015).

**TABLE 2:** The degree of crystallinity (X_c_,%); and melting (T_ms_) and glass transition (T_g_,°C) temperatures of the soft segments. The melting temperatures (T_mh_,°C) of the hard segments. Crystallization onset temperatures (T_onset_,°C).

Sample	X_c_ (%)	T_ms_ (°C)	T_mh_ (°C)	T_g_ (°C)	T_onset_ (°C)
PCL	69.76	53.2	-	−65.6	30.8
PU	31.35	49.1	162.3	−56.8	22.3
PU-0.1	35.17	48.9	163.6	−56.4	20.2
PU-0.5	29.56	47.2	168.7	−55.9	19.4
PU-1	28.86	44.9	171.6	−55.4	12.9
PU-2	25.18	42.4	172.7	−55.1	12.5

### Thermal and mechanical properties of SMPU/i-MWCNT composites

Figure 3C shows DSC curves of PCL, SMPU, and SMPU/i-MWCNT composites with various i-MWCNT contents. The results are also shown in Table 2. A PCL crystal melting peak is observed at approximately 50°C in all groups. In addition, an absorption peak is detected from SMPU and the SMPU/i-MWCNT composites at approximately 170°C. This peak appears because of melting of hard-segment crystals or long-range ordered dissociation of the hard segment amorphous state (Sofla et al., 2019; Ren et al., 2019). As the i-MWCNT content increases, the glass transition (T_g_) temperatures of the SMPU/i-MWCNT composites increase, the crystallinity (X_c_) of the soft segment and the crystallization onset temperatures (T_onset_) are reduced. This may be attributed to the limiting effect of nanoparticles on soft-segment fluidity. The decrease in crystallinity shows that i-MWCNTs can inhibit SMPU crystallization rather than nucleation, and thus may lead to a decrease in the initial SMPU crystallization temperature. Due to the appearance of nanoparticles, the crystals within the hard segment in SMPU are imperfect and the thicknesses of the crystal flakes are reduced. This reduces the melting temperature of the soft segment (T_ms_). The melting temperature (T_mh_) and crystallization enthalpy of the hard segment increase because the addition of i-MWCNTs reduces the degree of microphase separation of PU and they can interact with hard-segment formation (Hashmi et al., 2015; Nouri et al., 2020).

The first cooling step of the DSC test was used to study the crystallization behaviors of the SMPU and SMPU/i-MWCNT composites. The relative crystallinities of the samples shift to lower temperatures as the i-MWCNT content increases (Figure 3D). This indicates that i-MWCNT cannot be used as nucleating agent and that steric hindrance delays crystal formation (Kim et al., 2015; Sofla et al., 2019; Gorbunova et al., 2020). The TG curves of the SMPU and SMPU/i-MWCNT composites are shown in Figure 3E. The improved thermal stability characteristics of the SMPU/i-MWCNT composites relative to pure SMPU can be attributed to the good compatibility of i-MWCNT. The thermal decomposition temperature of SMPU/i-MWCNT increases with the i-MWCNT content because the interactions between i-MWCNT and the hard segment increase. This may promote crystallization of the hard-segment phase (Song et al., 2009; Mogha and Kaushik, 2021).

To study the effects of i-MWCNT addition on the mechanical properties of SMPU, tensile tests were performed on SMPU and SMPU/i-MWCNT composites with various i-MWCNT contents (Figure 3F). Because of weak interfacial interactions between i-MWCNT and the polyurethane chains, the elongation at break decreases gradually as the i-MWCNT content increases. The elongation at break drops sharply when the i-MWCNT content exceeds 0.1 wt%. High i-MWCNT contents may lead to partial agglomeration in the samples, resulting in internal stress defects and easy fracture during stretching. However, by analyzing the tensile strength under low strain, one can find that the tensile strengths of SMPU/i-MWCNT composites increase with the i-MWCNT content. This is because the load from an external force can be transferred from the matrix to the nanofiller effectively, reducing the load on the polymer chains. Thus, the i-MWCNTs play a reinforcing role (Mahapatra et al., 2014; Hashmi et al., 2015; Sofla et al., 2019).

**FIGURE 4:**
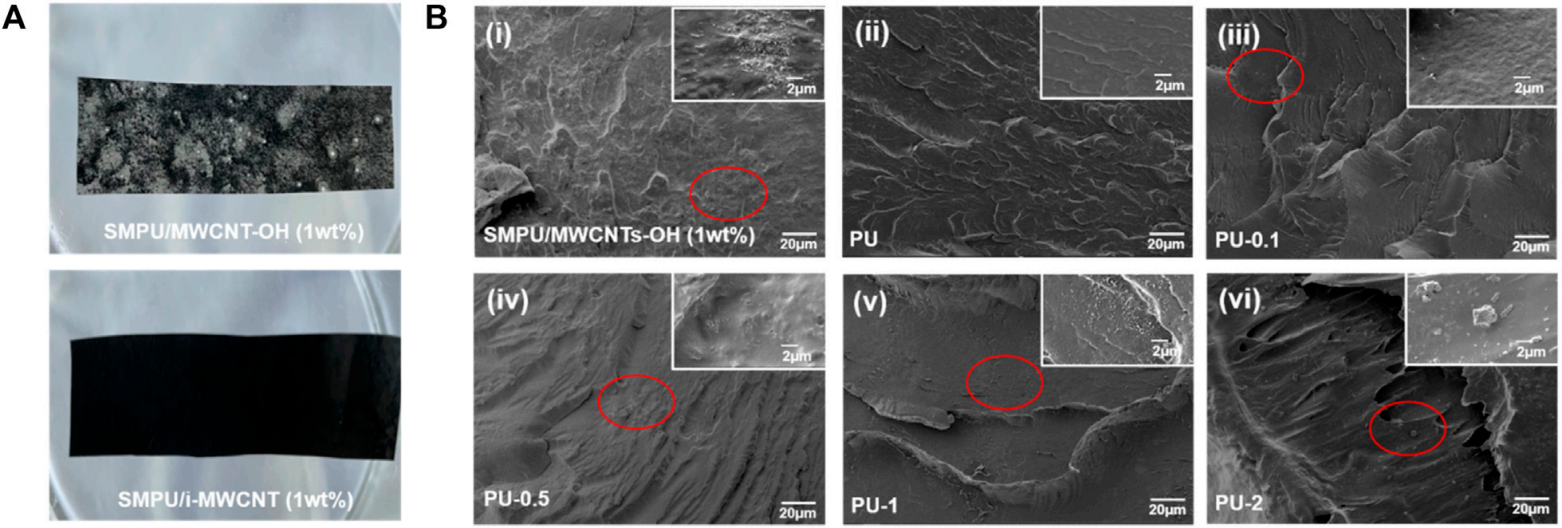
**(A)** The gross appearances of SMPU/MWCNT-OH and SMPU/i-MWCNT composites with MWCNT-OH or i-MWCNT contents of 1 wt%. **(B)** SEM images of **(I)** SMPU/MWCNT-OH (with 1 wt% MWCNT-OH) and SMPU/i-MWCNT composites with (i)-MWCNT contents of (ii) 0%, (iii) 0.1%, (iv) 0.5%, (v) 1%, and (vi) 2%.

Figure 4A shows the typical gross appearances of the SMPU/MWCNT-OH and SMPU/i-MWCNT composites. Unmodified MWCNT-OH exhibits poor dispersibility in the polymer matrix, while functionalized i-MWCNT has good dispersibility and no macroscopic agglomeration is observed. To analyze the distribution of carbon nanotubes in SMPU accurately, the fracture surfaces of pure SMPU and the composites were observed via SEM. Figure 4Bi shows that the nanoparticles agglomerate across a large area of the polymer when unmodified MWCNT-OH is added. However, such agglomeration is not observed in Figure 4B. This indicates that functionally modified i-MWCNT has better dispersion properties in the polymer matrix than its unmodified counterpart. In addition, when the i-MWCNT content of a composite is too low, the nanotubes disperse individually within the polymer and become wrapped by non-conductive SMPU, resulting in composites with no conductivity. A small amount of agglomeration occurs when the i-MWCNT content exceeds 0.5 wt%. This is a critical factor in determining whether the polymer has electrical properties (Moghim et al., 2018; Bi et al., 2020).

### Conductive, electrothermal, and shape memory effects

**FIGURE 5:**
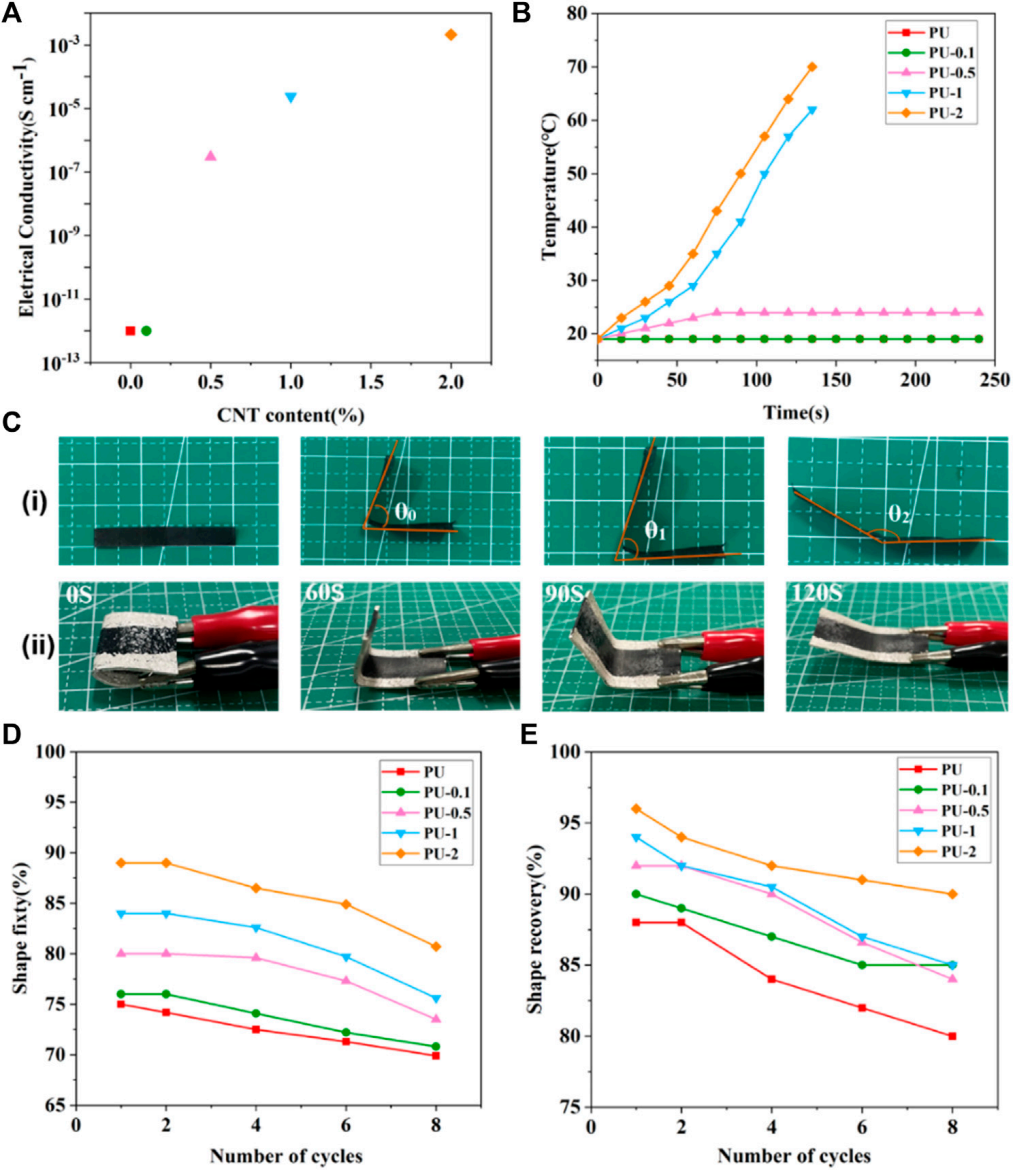
**(A)** Conductivity and **(B)** temperature changes among SMPU/i-MWCNT composites at a voltage of 30 eV. **(C)** Photos of (i) a shape deformation-recovery cycle and (ii) the shape recovery behaviors of SMPU/i-MWCNT composites with 1 wt% i-MWCNT content at a voltage of 30 eV. Quantitative analysis of the shape memory **(D)** fixation and **(E)** recovery rates over various shape strain-recovery cycles for SMPU/i-MWCNT composites with various i-MWCNT contents at temperatures below 60°C.

Figure 5A shows the electrical conductivities of SMPU/i-MWCNT composites with various i-MWCNT contents. When the i-MWCNT content is 0.1 wt%, the conductivity of the resulting composite is 10^−12^ S cm^−1^, which is similar to that of pure SMPU. Such a composite is not conductive and can be regarded as an insulator. However, when the i-MWCNT content is 0.5 wt%, the conductivity of the resulting composite exceeds 10^−7^ S cm^−1^. This indicates the formation of a conductive path. Therefore, it can be determined that 0.5 wt% is the approximate i-MWCNT conductance threshold content. Under this condition, functionally modified i-MWCNTs exhibit enhanced adhesion to and reduce the tunneling barrier formed by the SMPU. The manner in which the temperatures of SMPU/i-MWCNT composites change under a voltage of 30 eV over time is shown in Figure 5B. When the i-MWCNT concentration exceeds 1 wt%, the sample temperature can increase beyond 60°C within 140 s (Hajializadeh et al., 2017; Sang et al., 2019).

The shape memory effect of SMPU is related to its soft and hard segments. The PCL soft phase that can be crystallized serves as the soft segment and is responsible for maintaining the temporary shape. In contrast, the hard segment mainly controls the permanent shape of the sample. Table 3 shows the shape memory properties of SMPU and several SMPU/i-MWCNT composites with various i-MWCNT contents. The shape fixation and recovery rates (at 60 °C) increase gradually as the i-MWCNT content increases. However, the SMPU/i-MWCNT composite exhibits shape recovery abilities under a voltage of 30 eV within 120 s only when the i-MWCNT content exceeds 1 wt%. The composites with i-MWCNT contents of 1 wt% and 2 wt% exhibit shape recovery rates of 81 and 85%, respectively. A SMPU/i-MWCNT composite shape strain-recovery cycle is shown in Figure 5C, and is consistent with the quantitative analysis results. The reinforced electroactive shape memory effects may be attributed to addition of i-MWCNT, which reduces the degree of microphase separation within the SMPU and effectively increases the extent of interfacial interaction with SMPU in the composites (Khasraghi et al., 2019).

**TABLE 3:** Shape memory properties of SMPU and SMPU/i-MWCNT composites with various i-MWCNT contents.

Sample	R_f_ (%)	R_r_ (%) (Thermal: 60°C)	R_r_ (%) (Electrical: 30 eV)
PU	75	86	——
PU-0.1	76	90	——
PU-0.5	80	91	——
PU-1	84	93	81
PU-2	89	96	85

To investigate the shape memory effects within the SMPU/i-MWCNT composites further, the shape memory fixation (R_f_) and recovery (R_r_) rates of SMPU samples without and with various i-MWCNT contents were tested using multiple shape deformation-recovery cycles. As shown in Figures 5D,E, the shape memory fixation and recovery rates decrease in all groups. This is because the prepared SMPU is a linear polymer with intermolecular interactions that rely only on hydrogen bonding between hard and soft segments. The internal network points become damaged and thereby affect the shape memory performance after repeated shape deformation-recovery cycles. However, it is notable that the shape memory fixation and recovery rates of the composites with i-MWCNT contents greater than 1 wt% remain higher than 80 and 90%, respectively, even after up to eight shape deformation-recovery cycles.

## Conclusion

In this study, hydroxylated MWCNTs were functionalized with octadecyl isocyanate to produce i-MWCNT without affecting the chemical structures of the nanotubes. The dispersion performance characteristics of the i-MWCNTs in organic reagents and the polymer matrix improved significantly. In addition, an electroactive shape memory composite (SMPU/i-MWCNT) was developed by blending i-MWCNT with shape memory polyurethane. The addition of i-MWCNT reduced the crystallinity of SMPU and decreased the hydrogen bond index and melting temperature of the SMPU soft segment while increasing the thermal decomposition temperatures of the composites. The SMPU/i-MWCNT composites exhibited substantial conductivity. This conductivity increased with the i-MWCNT content. When the i-MWCNT content exceeded 1 wt%, the temperature of the composite could increase beyond 60°C within 140 s and the temporary structure could be restored to its initial state within 120 s under a voltage of 30 eV. The shape memory effect was maintained effectively after eight cycles of testing. Therefore, the functionalized CNTs exhibit excellent potential for the development of electroactive shape memory composites, which may be used in flexible electronics and other fields.

## Data Availability

The original contributions presented in the study are included in the article/supplementary material, further inquiries can be directed to the corresponding author.

## References

[B1] Babaie A. Rezaei M. Sofla R. L. M. (2019). Investigation of the effects of polycaprolactone molecular weight and graphene content on crystallinity, mechanical properties and shape memory behavior of polyurethane/graphene nanocomposites. J. Mech. Behav. Biomed. Mat. 96, 53–68. 10.1016/j.jmbbm.2019.04.034 31029995

[B2] Behl M. Lendlein A. (2007). Shape-memory polymers. Mater. Today 10, 20–28. 10.1016/s1369-7021(07)70047-0

[B3] Bi H. Ye G. Yang H. Sun H. Ren Z. Guo R. (2020). Near infrared-induced shape memory polymer composites with dopamine-modified multiwall carbon nanotubes via 3D-printing. Eur. Polym. J. 136, 109920. 10.1016/j.eurpolymj.2020.109920

[B4] Chazot C. a. C. Hart A. J. (2019). Understanding and control of interactions between carbon nanotubes and polymers for manufacturing of high-performance composite materials. Compos. Sci. Technol. 183, 107795. 10.1016/j.compscitech.2019.107795

[B5] Deng J. Zhang X. Wang K. Zou H. Zhang Q. Fu Q. (2007). Synthesis and properties of poly(ether urethane) membranes filled with isophorone diisocyanate-grafted carbon nanotubes. J. Membr. Sci. 288, 261–267. 10.1016/j.memsci.2006.11.033

[B6] Du C. Liu J. Fikhman D. A. Dong K. S. Monroe M. B. B. (2022). Shape memory polymer foams with phenolic acid-based antioxidant and antimicrobial properties for traumatic wound healing. Front. Bioeng. Biotechnol. 10, 809361. 10.3389/fbioe.2022.809361 35252129 PMC8893234

[B7] Eyvazzadeh Kalajahi A. Rezaei M. Abbasi F. Mir Mohamad Sadeghi G. (2017). The effect of chain extender type on the physical, mechanical, and shape memory properties of poly(ε-caprolactone)-based polyurethane-ureas. Polymer-Plastics Technol. Eng. 56, 1977–1985. 10.1080/03602559.2017.1298797

[B8] Galindo B. Benedito A. Gimenez E. Compañ V. (2016). Comparative study between the microwave heating efficiency of carbon nanotubes versus multilayer graphene in polypropylene nanocomposites. Compos. Part B Eng. 98, 330–338. 10.1016/j.compositesb.2016.04.082

[B9] Gao H. Li J. Zhang F. Liu Y. Leng J. (2019). The research status and challenges of shape memory polymer-based flexible electronics. Mat. Horiz. 6, 931–944. 10.1039/c8mh01070f

[B10] Gorbunova M. A. Zaitsev V. Grishchuk A. A. Badamshina E. R. (2020). “Roles of phase separation and crystallization in the structure formation,” in Russian chemical bulletin. International Edition.

[B11] Hager M. D. Bode S. Weber C. Schubert U. S. (2015). Shape memory polymers: Past, present and future developments. Prog. Polym. Sci. 49-50, 3–33. 10.1016/j.progpolymsci.2015.04.002

[B12] Hajializadeh S. Barikani M. Bellah S. M. (2017). Synthesis and characterization of multiwall carbon nanotube/waterborne polyurethane nanocomposites. Polym. Int. 66, 1074–1083. 10.1002/pi.5362

[B13] Hardy J. G. Palma M. Wind S. J. Biggs M. J. (2016). Responsive biomaterials: Advances in materials based on shape-memory polymers. Adv. Mat. 28, 5717–5724. 10.1002/adma.201505417 27120512

[B14] Hashmi S. a. R. Prasad H. C. Abishera R. Bhargaw H. N. Naik A. (2015). Improved recovery stress in multi-walled-carbon-nanotubes reinforced polyurethane. Mater. Des. 67, 492–500. 10.1016/j.matdes.2014.10.062

[B15] Hu J. Zhu Y. Huang H. Lu J. (2012). Recent advances in shape–memory polymers: Structure, mechanism, functionality, modeling and applications. Prog. Polym. Sci. 37, 1720–1763. 10.1016/j.progpolymsci.2012.06.001

[B16] Ke K. Solouki Bonab V. Yuan D. Manas-Zloczower I. (2018). Piezoresistive thermoplastic polyurethane nanocomposites with carbon nanostructures. Carbon 139, 52–58. 10.1016/j.carbon.2018.06.037

[B17] Khasraghi S. S. Shojaei A. Sundararaj U. (2019). Bio-based UV curable polyurethane acrylate: Morphology and shape memory behaviors. Eur. Polym. J. 118, 514–527. 10.1016/j.eurpolymj.2019.06.019

[B18] Kim Y. Kwon S.-M. Kim D.-Y. Kim H.-S. Jin H.-J. (2009). Dispersity and stability measurements of functionalized multiwalled carbon nanotubes in organic solvents. Curr. Appl. Phys. 9, e100–e103. 10.1016/j.cap.2008.12.039

[B19] Kim J. T. Jeong H. J. Park H. C. Jeong H. M. Bae S. Y. Kim B. K. (2015). Electroactive shape memory performance of polyurethane/graphene nanocomposites. React. Funct. Polym. 88, 1–7. 10.1016/j.reactfunctpolym.2015.01.004

[B20] Kim J. Ko H. U. Kim H. C. (2021). Refractive index change of cellulose nanocrystal-based electroactive polyurethane by an electric field. Front. Bioeng. Biotechnol. 9, 606008. 10.3389/fbioe.2021.606008 33634083 PMC7901916

[B21] Laza J. M. Veloso A. Vilas J. L. (2020). Tailoring new bisphenol a ethoxylated shape memory polyurethanes. J. Appl. Polym. Sci. 138, 49660. 10.1002/app.49660

[B22] Leng J. Lan X. Liu Y. Du S. (2011). Shape-memory polymers and their composites: Stimulus methods and applications. Prog. Mater. Sci. 56, 1077–1135. 10.1016/j.pmatsci.2011.03.001

[B23] Liu W. Zhao Y. Wang R. Li J. Li J. Luo F. (2017). Post-crosslinked polyurethanes with excellent shape memory property. Macromol. Rapid Commun. 38, 1700450. 10.1002/marc.201700450 29083102

[B24] Liu J. Chen Z. Hu C. Yang W. Wang J. Xu W. (2021). Fluorescence visualization directly monitors microphase separation behavior of shape memory polyurethanes. Appl. Mater. Today 23, 100986. 10.1016/j.apmt.2021.100986

[B25] Lopes M. C. Ribeiro H. Gonçalves Santos M. C. Seara L. M. Queiroz Ferreira F. L. Lavall R. L. (2017). High performance polyurethane composites with isocyanate-functionalized carbon nanotubes: Improvements in tear strength and scratch hardness. J. Appl. Polym. Sci. 134. 10.1002/app.44394

[B26] Mahapatra S. S. Yadav S. K. Yoo H. J. Ramasamy M. S. Cho J. W. (2014). Tailored and strong electro-responsive shape memory actuation in carbon nanotube-reinforced hyperbranched polyurethane composites. Sensors Actuators B Chem. 193, 384–390. 10.1016/j.snb.2013.12.006

[B27] Mogha A. Kaushik A. (2021). Functionalized multiwall carbon nanotubes to enhance dispersion in castor oil-based polyurethane nanocomposites. Fullerenes, Nanotub. Carbon Nanostructures 29, 907–914. 10.1080/1536383x.2021.1915295

[B28] Moghim M. H. Zebarjad S. M. Eqra R. (2018). Experimental and modeling investigation of shape memory behavior of polyurethane/carbon nanotube nanocomposite. Polym. Adv. Technol. 29, 2496–2504. 10.1002/pat.4361

[B29] Nejad S. Rezaei M. Bagheri M. (2019). Polyurethane/nitrogen-doped graphene quantum dot (N-gqd) nanocomposites: Synthesis, characterization, thermal, mechanical and shape memory properties. Polymer-Plastics Technol. Mater. 59, 398–416. 10.1080/25740881.2019.1647243

[B30] Nouri N. Rezaei M. Mayan Sofla R. L. Babaie A. (2020). Synthesis of reduced octadecyl isocyanate-functionalized graphene oxide nanosheets and investigation of their effect on physical, mechanical, and shape memory properties of polyurethane nanocomposites. Compos. Sci. Technol. 194, 108170. 10.1016/j.compscitech.2020.108170

[B31] Park S. Moon J. Kim B. Cho M. (2021). Multi-scale coarse-grained molecular dynamics simulation to investigate the thermo-mechanical behavior of shape-memory polyurethane copolymers. Polymer 213, 123228. 10.1016/j.polymer.2020.123228

[B32] Qu M. Wang H. Chen Q. Wu L. Tang P. Fan M. (2022). A thermally-electrically double-responsive polycaprolactone – Thermoplastic polyurethane/multi-walled carbon nanotube fiber assisted with highly effective shape memory and strain sensing performance. Chem. Eng. J. 427, 131648. 10.1016/j.cej.2021.131648

[B33] Rahmat M. Das K. Hubert P. (2011). Interaction stresses in carbon nanotube-polymer nanocomposites. ACS Appl. Mat. Interfaces 3, 3425–3431. 10.1021/am200652f 21805984

[B34] Raza T. Qu L. Khokhar W. A. Andrews B. Ali A. Tian M. (2021). Progress of wearable and flexible electrochemical biosensors with the aid of conductive nanomaterials. Front. Bioeng. Biotechnol. 9, 761020. 10.3389/fbioe.2021.761020 34881233 PMC8645837

[B35] Ren D. Chen Y. Li H. Rehman H. U. Cai Y. Liu H. (2019). High-efficiency dual-responsive shape memory assisted self-healing of carbon nanotubes enhanced polycaprolactone/thermoplastic polyurethane composites. Colloids Surfaces A Physicochem. Eng. Aspects 580, 123731. 10.1016/j.colsurfa.2019.123731

[B36] Rosales C. a. G. Duarte M. F. G. Kim H. Chavez L. Hodges D. Mandal P. (2018). 3D printing of shape memory polymer (SMP)/carbon black (CB) nanocomposites with electro-responsive toughness enhancement. Mat. Res. Express 5, 065704. 10.1088/2053-1591/aacd53

[B37] Sahoo N. G. Rana S. Cho J. W. Li L. Chan S. H. (2010). Polymer nanocomposites based on functionalized carbon nanotubes. Prog. Polym. Sci. 35, 837–867. 10.1016/j.progpolymsci.2010.03.002

[B38] Salam M. A. Burk R. (2017). Synthesis and characterization of multi-walled carbon nanotubes modified with octadecylamine and polyethylene glycol. Arabian J. Chem. 10, S921–S927. 10.1016/j.arabjc.2012.12.028

[B39] Sang Z. Ke K. Manas-Zloczower I. (2019). Effect of carbon nanotube morphology on properties in thermoplastic elastomer composites for strain sensors. Compos. Part A Appl. Sci. Manuf. 121, 207–212. 10.1016/j.compositesa.2019.03.007

[B40] Sofla R. M. L Rezaei M. Babaie A. Nasiri M. (2019). Preparation of electroactive shape memory polyurethane/graphene nanocomposites and investigation of relationship between rheology, morphology and electrical properties. Compos. Part B Eng. 175, 107090. 10.1016/j.compositesb.2019.107090

[B41] Song W.-H. Ni Q.-P. Zheng Z. Tian L.-Y. Wang X.-L. (2009). The preparation of biodegradable polyurethane/carbon nanotube composite based onin situcross-linking. Polym. Adv. Technol. 20, 327–331. 10.1002/pat.1271

[B42] Sun L. Gao X. Wu D. Guo Q. (2020). Advances in physiologically relevant actuation of shape memory polymers for biomedical applications. Polym. Rev. 61, 280–318. 10.1080/15583724.2020.1825487

[B43] Sun J. Peng B. Lu Y. Zhang X. Wei J. Zhu C. (2022). A photoorganizable triple shape memory polymer for deployable devices. Small 18, e2106443. 10.1002/smll.202106443 34918481

[B44] Tamore M. S. Ratna D. Mishra S. Shimpi N. G. (2019). Effect of functionalized multi-walled carbon nanotubes on physicomechanical properties of silicone rubber nanocomposites. J. Compos. Mater. 53, 3157–3168. 10.1177/0021998319827080

[B45] Umair M. M. Zhang Y. Zhang S. Jin X. Tang B. (2019). A novel flexible phase change composite with electro-driven shape memory, energy conversion/storage and motion sensing properties. J. Mat. Chem. A Mat. 7, 26385–26392. 10.1039/c9ta09088f

[B46] Ur Rehman H. Chen Y. Hedenqvist M. S. Li H. Xue W. Guo Y. (2018). Self-Healing shape memory PUPCL copolymer with high cycle life. Adv. Funct. Mat. 28, 1704109. 10.1002/adfm.201704109

[B47] Wen Z. Mcbride M. K. Zhang X. Han X. Martinez A. M. Shao R. (2018). Reconfigurable LC elastomers: Using a thermally programmable monodomain to access two-way free-standing multiple shape memory polymers. Macromolecules 51, 5812–5819. 10.1021/acs.macromol.8b01315

[B48] Xia Y. He Y. Zhang F. Liu Y. Leng J. (2021). A review of shape memory polymers and composites: Mechanisms, materials, and applications. Adv. Mat. 33, e2000713. 10.1002/adma.202000713 32969090

[B49] Xie H. Shao J. Ma Y. Wang J. Huang H. Yang N. (2018). Biodegradable near-infrared-photoresponsive shape memory implants based on black phosphorus nanofillers. Biomaterials 164, 11–21. 10.1016/j.biomaterials.2018.02.040 29477708

[B50] Xu X. Fan P. Ren J. Cheng Y. Ren J. Zhao J. (2018). Self-healing thermoplastic polyurethane (TPU)/polycaprolactone (PCL)/multi-wall carbon nanotubes (MWCNTs) blend as shape-memory composites. Compos. Sci. Technol. 168, 255–262. 10.1016/j.compscitech.2018.10.003

[B51] Zahedi F. Amraee I. A. (2018). Carboxylated multiwalled carbon nanotubes effect on dynamic mechanical behavior of soft films composed of multilayer polymer structure. Polymer 151, 187–196. 10.1016/j.polymer.2018.07.044

[B52] Zhang Z.-X. Dou J.-X. He J.-H. Xiao C.-X. Shen L.-Y. Yang J.-H. (2017). Electrically/infrared actuated shape memory composites based on a bio-based polyester blend and graphene nanoplatelets and their excellent self-driven ability. J. Mat. Chem. C Mat. 5, 4145–4158. 10.1039/c7tc00828g

[B53] Zhao C. Ji L. Liu H. Hu G. Zhang S. Yang M. (2004). Functionalized carbon nanotubes containing isocyanate groups. J. Solid State Chem. 177, 4394–4398. 10.1016/j.jssc.2004.09.036

[B54] Zheng Y. Qin J. Shen J. Guo S. (2020). Controllable distribution of conductive particles in polymer blends via a bilayer structure design: A strategy to fabricate shape-memory composites with tunable electro-responsive properties. J. Mat. Chem. C Mat. 8, 9593–9601. 10.1039/d0tc01854f

[B55] Zhu Y. Hu J. Choi K.-F. Meng Q. Chen S. Yeung K.-W. (2008). Shape memory effect and reversible phase crystallization process in SMPU ionomer. Polym. Adv. Technol. 19, 328–333. 10.1002/pat.1001

